# Ergothioneine as a Natural Antioxidant Against Oxidative Stress-Related Diseases

**DOI:** 10.3389/fphar.2022.850813

**Published:** 2022-03-18

**Authors:** Tong-Tong Fu, Liang Shen

**Affiliations:** ^1^ Institute of Biomedical Research, Shandong University of Technology, Zibo, China; ^2^ Shandong Provincial Research Center for Bioinformatic Engineering and Technique, Zibo Key Laboratory of New Drug Development of Neurodegenerative Diseases, School of Life Sciences and Medicine, Shandong University of Technology, Zibo, China

**Keywords:** L-ergothioneine, antioxidant, oxidative stress, action mechanism, disease

## Abstract

L-Ergothioneine (EGT) is a natural antioxidant derived from microorganisms, especially in edible mushrooms. EGT is found to be highly accumulated in tissues that are susceptible to oxidative damage, and it has attracted extensive attention due to its powerful antioxidant activity and the tight relationships of this natural product with various oxidative stress-related diseases. Herein, we 1) introduce the biological source and *in vivo* distribution of EGT; 2) review the currently available evidence concerning the relationships of EGT with diabetes, ischemia-reperfusion injury-related diseases like cardiovascular diseases and liver diseases, neurodegenerative diseases, and other diseases pathogenically associated with oxidative stress; 3) summarize the potential action mechanisms of EGT against these diseases; 4) discuss the advantages of EGT over other antioxidants; and 5) also propose several future research perspectives for EGT. These may help to promote the future application of this attractive natural antioxidant.

## 1 Introduction

**FIGURE 1:**
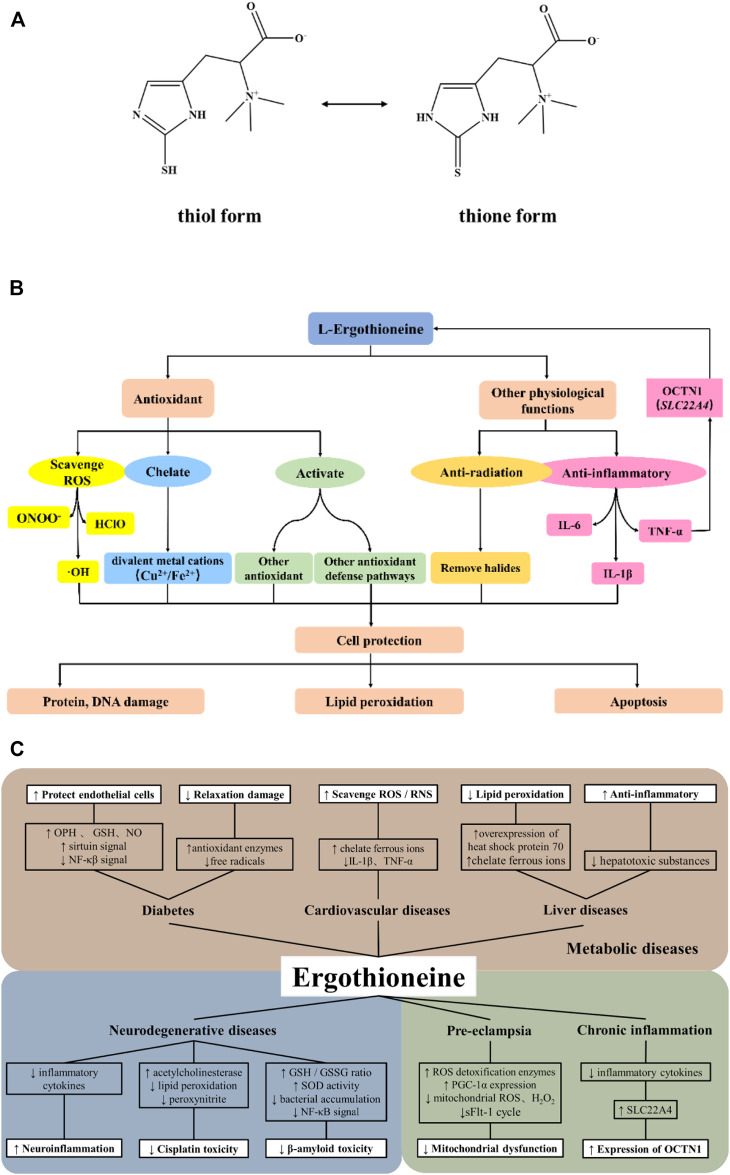
Antioxidant properties and potential action mechanisms of ergothioneine. **(A)**. Chemical structure of ergothioneine; **(B)**. The main antioxidant properties and cytoprotective effects of ergothioneine; **(C)**. Potential mechanisms underlying the relationships between ergothioneine and oxidative stress-related disease conditions.

L-Ergothioneine (EGT) is a natural thiourea derivative of histidine and exists in two forms: thiol and thione forms (Figure 1A). It was first discovered by Tanret in *Claviceps purpurea* in 1909 (Tanret, 1909). As a low molecular weight (LMW) thiol, the presence of the sulfhydryl group endows it with a wide range of beneficial effects such as anti-oxidation, anti-inflammatory, and detoxification, thereby preventing biomolecular damage (Sao Emani et al., 2019). Therefore, it exhibits possible protective effects in various oxidative stresses of organisms. Its unique physical and chemical properties (these are also explained in detail below) have made it a research hotspot since its discovery. It has been widely used as a dietary supplement and cosmetic additive (Han et al., 2021). A novel food, synthetic EGT, was proved to be safe under the expected use conditions by the European Food Safety Authority Panel on Dietetic Products, Nutrition and Allergies, and is recommended as a food supplement at the recommended dosage of 30 mg/day for adults and 20 mg/day for children (Turck et al., 2016; Turck et al., 2017).

This paper mainly discusses the basic antioxidant properties of EGT in animals and its close relationship with oxidative stress diseases, and summarizes the possible therapeutic or protective mechanisms of EGT, which may provide implications for future research directions and promote further application of EGT.

### 1.1 Biological Sources of L-Ergothioneine

The synthesis of LMW thiol compounds is widespread in prokaryotic and eukaryotic organisms (Sao Emani et al., 2019). But so far, EGT is synthesized only by certain microorganisms such as *Actinomycetales* (Genghof, 1970), *Mycobacteria* (Genghof and Vandamme, 1964; Sao Emani et al., 2013), *Cyanobacteria* (Pfeiffer et al., 2011; Narainsamy et al., 2016; Liao and Seebeck, 2017), and other bacteria, and *Neuropora crassa* (Hu et al., 2014), non-yeast fungi (related to asexual spores) (Genghof, 1970). Especially edible fungi, mushrooms, have been proven to be the highest dietary source of EGT, accounting for about 95% of dietary intake (Ito et al., 2011; Weigand-Heller et al., 2012; Kalaras et al., 2017). The EGT content varies between different mushroom species. In previous test reports, the measurement results were slightly different due to differences in assay methods and conditions, but generally *Pleurotus* genus (including *P. citrinopileatus*, *P. ostreatus*, *P. salmoneostramisus*, *etc.*) contained high levels of EGT [0.22–3.94 mg/g dry weight (dw)], followed by *Lentinus* genus, *Grifola* genus and *Agrocybe* genus (∼3 mg/g dw) (Dubost et al., 2007; Ito et al., 2011; Chen et al., 2012; Lo et al., 2012; Kalaras et al., 2017; Tsiantas et al., 2021). This may be related to the content and bioavailability of the material bases for EGT synthesis, such as histidine, cysteine, and methionine, contained in different mushroom species (Tsiantas et al., 2021). It has been found that the EGT level of porcini in *Boletus edulis* can be as high as 7.27 mg/g dw (Kalaras et al., 2017).

There may be a high correlation between the synthesis of EGT and glutathione (GSH). The GSH synthesis genes *gshA* and *gshB* could be used in the synthesis process of EGT, at the same time, EGT could also promote the synthesis of GSH through the Nrf2/ARE pathway (Ito et al., 2011; Narainsamy et al., 2016; Kalaras et al., 2017). It seems to indicate that these two antioxidants may interact to protect organisms during oxidative stress. However, in the seminal plasma of boar, it was found that EGT had nothing to do with the level of GSH, suggesting that EGT may not depend on the redox cycle of GSH at least in this extracellular environment (Nikodemus et al., 2011). It is therefore speculated that the relationship between EGT and GSH may be related to the different oxidative stress states of the environment, which requires more experimental verification. Interestingly, although some bacteria, such as *Actinobacteria*, *Mycobacterium*, are also present in large quantities in the intestinal tract, EGT synthesis has never been found in animals. The mystery has not been solved so far. (Cheah and Halliwell, 2021) proposed that the EGT contained in the intestinal flora may be absorbed and accumulated from the external environment, because the EGT precursors labeled by isotope did not show signs of synthesis (Cheah and Halliwell, 2021). In view of the close the relationships between intestinal microbes and health and diseases, the correlation between intestinal microbes and EGT tissue levels becomes particularly important (Gamage et al., 2018; Cheah and Halliwell, 2021), which merits further investigation.

### 1.2 *In vivo* Distribution of L-Ergothioneine

Although EGT cannot be synthesized by animals, it has been shown to be widely taken up and transported into cells and tissues by the specific carnitine/organic cation transporter OCTN1 [also known as ergothioneine transporter (ETT)] on the cell membrane from food (such as mushrooms, grains, internal organs) (Gründemann et al., 2005; Ey et al., 2007; Nakamura et al., 2008; Gründemann, 2012). Food-derived EGT can be effectively absorbed by OCTN1 in the intestinal tract with the lowest metabolism and quickly distributed to cells and tissues in contact with blood (Pfeiffer et al., 2011; Gründemann, 2012; Turck et al., 2016). OCTN1 gene (*SLC22A4*) knockout (Kato et al., 2010; Pfeiffer et al., 2015) and overexpression (Jong et al., 2011; Tschirka et al., 2018) studies demonstrated the complete dependence of EGT on OCTN1. Due to the different expression of OCTN1 in different tissues, the content of EGT in different tissues also varies greatly, and it is not accumulated in cells and tissues lacking OCTN1 (Melville et al., 1954; Gründemann et al., 2005; Kato et al., 2010; Gründemann, 2012; Tang et al., 2018). According to previous studies, basal EGT levels are the highest in liver and erythrocytes (Heath, 1953; Kato et al., 2010; Weigand-Heller et al., 2012; Halliwell et al., 2018; Tang et al., 2018), and also massively accumulated in intestines, semen, testis, bone marrow, kidney, spleen, lung, eye, and the brain in human body (∼0.1–1 mM) (Heath, 1953; Melville et al., 1954; Mayumi et al., 1978; Kawano et al., 1982b; Shires et al., 1997; Gründemann et al., 2005; Kato et al., 2010; Halliwell et al., 2018; Tang et al., 2018). Especially in the tissues and organs that are susceptible to oxidative stress and inflammation, EGT interestingly maintains a high level (Kato et al., 2010; Halliwell et al., 2018). These evidence supports that EGT should be an important biologically active substance in the body. There is no significant correlation between EGT and gender, while studies on the correlation between EGT and age showed that during growth, liver and red blood cell EGT levels increased with age (Mackenzie and Mackenzie, 1957; Kawano et al., 1982b; Kumosani, 2001). Among the middle-aged and elderly, the levels of whole blood and plasma EGT decrease with age, which is considered to be related to changes of dietary habits or *SLC22A4* gene expression (Sotgia et al., 2014; Cheah et al., 2016a). Although there are no symptoms related to EGT deficiency in the healthy model (Kawano et al., 1982b; Kato et al., 2010; Pfeiffer et al., 2015), which may be due to the existence of compensatory defense pathways (Cheah and Halliwell, 2021), the plasma level of patients with mild cognitive impairment is significantly reduced (Cheah et al., 2016a). This phenomenon implies that EGT deficiency may be related to neuropathy and aging, which may increase the risk of aging-related oxidative stress diseases in the elderly. In 2017, a study of oral EGT and its uptake and pharmacokinetics in healthy volunteers confirmed for the first time that EGT could be strongly absorbed and retained in the body, and showed a significant increase in plasma and whole blood concentrations. On the contrary, the biomarkers of oxidative damage and inflammation showed a downward trend (Cheah et al., 2017). This further indicates that EGT could play a physiological role as an important antioxidant in human body.

### 1.3 The Antioxidant Properties and Cytoprotective Effects of L-Ergothioneine

It is well known that EGT is an effective physiological antioxidant (Akanmu et al., 1991; Chaudière and Ferrari-Iliou, 1999; Cheah and Halliwell, 2012; Gründemann, 2012; Halliwell et al., 2018) and its antioxidant effect is mainly manifested by several following mechanisms (Figure 1B). Firstly, EGT can not only prevent the formation of free radicals such as OH, but also directly scavenge free radicals and reactive oxygen species (ROS) such as hypochlorite acid (HClO) and peroxynitrite (Akanmu et al., 1991; Aruoma et al., 1997; Franzoni et al., 2006; Cheah and Halliwell, 2012; Asahi et al., 2016). EGT also shows a higher rate of inactivating singlet oxygen than other thiols under physiological pH (Rougee et al., 1988; Stoffels et al., 2017). Secondly, EGT can interact with other natural antioxidant defense systems in the body, such as activating the intracellular antioxidant pathway involving MAPKs and regulating the levels of peroxidases and antioxidant enzymes like superoxide dismutases (Kawano et al., 1983; Chaudière and Ferrari-Iliou, 1999; Colognato et al., 2006). Thirdly, EGT chelates a variety of divalent metal cations, e.g., Fe, Cu, Zn, Ni, and Co. Unlike other thiol compounds, the chelation of EGT results in the formation of the redox-inactive ergothioneine-metal complex which constrains the reactivity of metal ions (Hanlon, 1971; Zhu et al., 2011). At the same time, EGT can also selectively inhibit the activity of some Zn- and Cu-requiring metalloenzymes, thus inhibiting the oxidation of these metal ions and preventing them from participating in the formation of ROS in the body (Paul and Snyder, 2010).

Owing to its antioxidant properties, EGT plays a powerful cytoprotective role in some important cells and tissues (Paul and Snyder, 2010; Rajesh and Dash, 2018). In the myocardium, EGT could reduce ferryl myoglobin by coupling GSH to prevent the accumulation of the hypervalent state of Fe (Arduini et al., 1990). In the erythrocyte, EGT could inhibit the peroxidation of the mixture of H_2_O_2_ and hemoglobin on arachidonic acid (Akanmu et al., 1991). EGT also has the ability to inhibit nitrite-induced oxyhemoglobin oxidation by scavenging nitrogen dioxide, which postpones or reverses the form of ferryl hemoglobin and methemoglobin (Spicer et al., 1951; Arduini et al., 1992). Moreover, it protects erythrocytes from neutrophils damage by removing HClO (Cheah and Halliwell, 2012). In addition, some studies have also demonstrated that OCTN1 can be expressed in large amounts in mitochondria, allowing EGT to enter mitochondria and inhibit the generation of free radicals and ROS in the electron transport chain (Lamhonwah and Tein, 2006; Paul and Snyder, 2010). Thus, EGT exerts the cytoprotective effect to protect DNA, proteins, lipids, and other components from oxidative damage and protect cells from ROS-induced apoptosis (Paul and Snyder, 2010; Halliwell et al., 2016).

## 2 Relationships of L-Ergothioneine With Oxidative Stress-Related Diseases

Oxidative stress and inflammation are key pathogenic factors in many diseases. High levels of EGT can be detected in many tissues or cells and accumulating studies support the preventive or therapeutic potentials of EGT in a series of oxidative stress-related diseases through antioxidation. Therefore, we summarize the relationships of EGT with several oxidative stress-related diseases, and discuss its possible action mechanisms (Figure 1C).

### 2.1 Diabetes

Firstly, type 2 diabetes is closely related to obesity and diet. Supplementing the diet with EGT-rich mushrooms may be beneficial to diabetics (Lam-Sidun et al., 2021). For example, in a dietary treatment for patients with early diabetes, eating standard white button mushroom (EGT 3.2 mg/100 g) daily for 16 weeks could reduce systemic oxidative stress and inflammatory markers (Calvo et al., 2016). In addition, oxidative stress-associated endothelial dysfunction during hyperglycemia is closely related to the pathogenesis of diabetes and its complications, and it is mediated by the sirtuin signal (Félétou and Vanhoutte, 2006; Albiero et al., 2014). The antioxidant effect of EGT has been proven to improve endothelial cell senescence and vascular relaxation damage caused by the pro-oxidative effect of hyperglycemia in diabetes (Lam-Sidun et al., 2021). The protective and antioxidant effects of EGT treatment on endothelial cells exposed to hyperglycemia and its possible dependent mechanisms were studied by establishing the model of hyperglycemia-induced endothelial cell toxicity and senescence. Its antioxidant effect might mainly work through interaction with other antioxidant defense systems in the body. On the one hand, EGT regulates other antioxidant pathways. For example, EGT could up-regulate sirtuin 1 and sirtuin 6 as well as down-regulate p66Shc and NF-κB *in vivo* (D’Onofrio et al., 2016). At the same time, EGT could also regulate other antioxidant enzymes like glutathione synthetase to increase the intracellular level of GSH, and finally inhibit the production of free radicals and ROS (Servillo et al., 2017b). On the other hand, a study *in vitro* explored the effect of EGT on rat vascular reactivity, and it was concluded that EGT could protect nitric oxide from damage and maintain its activity by reducing superoxide anions (Gokce and Arun, 2014). Thus, EGT can prevent cells damage from hyperglycemia-dependent oxidative stress, and then protect endothelial integrity and maintain endothelial cell and vascular function.

### 2.2 Ischemia-Reperfusion Injury

Ischemia-reperfusion (IR) injury triggers the massive production and accumulation of ROS and oxidative stress-mediated injury, which occurs in almost all organs, such as the heart, liver, and intestines (Zhou et al., 2018; Lam-Sidun et al., 2021). Many studies have proved the accumulation of EGT in different tissues during IR, which also suggests that supplementation of EGT may be a potential therapeutic approach for IR in various tissues (Tang et al., 2018).

#### 2.2.1 Cardiovascular Diseases

IR is considered to be an important pathogenic factor of atherosclerosis and other cardiovascular diseases (Libby et al., 2011). Transition metal iron and copper catalyze the formation of oxygen free radicals after myocardial IR (Spencer et al., 1998). It was observed that external intake of EGT could quickly accumulate in the heart through the blood (Tang et al., 2018), which might imply its possible protective effect on the cardiovascular system. A long-term follow-up survey study including 3,236 participants found that of 112 plasma metabolites, EGT had the most significant and positive association with healthy conscious food patterns. EGT could reduce the mortality of patients with cardiovascular diseases and the risk of healthy people suffering from cardiovascular diseases (Smith et al., 2020). Moreover, the EGT incubation experiment of myoglobin exposed to H_2_O_2_ and the EGT supplementation experiment on rat heart reperfusion after transient ischemia revealed the protective mechanisms of EGT on the heart. That is, EGT could couple GSH to reduce ferryl myoglobin, chelate transition metal iron to scavenge ROS/RNS and free radicals generated during IR, regulate proinflammatory cytokines like interleukin-1β (IL-1β) and tumor necrosis factor-α (TNF-α), and ultimately prevent myocardial injury (Arduini et al., 1990; Servillo et al., 2017a).

#### 2.2.2 Liver Diseases

The damage of IR to the liver is also extremely significant. Excessive accumulation and degeneration of lipids cause oxidative stress and inflammation in the liver, leading to chronic liver diseases such as nonalcoholic fatty liver disease (NAFLD). Research on the role of EGT in the liver is limited. It has been shown that the liver is the main site to accumulate EGT, and a previous animal study found that supplementation of EGT [70 mg/kg body weight (bw)] in rats for 7 days before injection of ferric-nitrilotriacetate could protect the liver from the injury of lipid peroxidation (Deiana et al., 2004). The indexes detection of the NAFLD animal model established by cholesterol showed that under stress, the liver could up-regulate the expression of OCTN1 to increase the uptake and accumulation of EGT, and there was a significant correlation between the liver level of EGT and cholesterol and iron, although there was no difference in the diet content of EGT (Cheah et al., 2016b). It was speculated that one of the possible protective mechanisms of EGT was to inhibit the Fenton reaction and reduce oxidative stress by chelating ferrous ions (Cheah et al., 2016b). Another mechanism of EGT was found by constructing the animal model of liver IR injury. It could promote the overexpression of heat shock protein 70 to improve liver injury tolerance and inhibit subsequent lipid peroxidation, thereby protecting the liver and improving the survival rate (Bedirli et al., 2004). Similarly, this protective mechanism was also affirmed in the experiment of EGT treatment on rat mesenteric IR (Sakrak et al., 2008).

### 2.3 Neurodegenerative Diseases

The prevalence of neurodegenerative diseases, including Alzheimer’s disease (AD), Parkinson’s disease, Huntington’s disease, and so on, in the elderly has increased steadily in recent years. It has been widely accepted that oxidative stress is one of the main causes of neurodegenerative diseases. For instance, oxidative stress caused by the deposition of β-amyloid protein (Aβ) plays an important role in the pathogenesis of AD (Schubert et al., 1995), and the neurotoxicity of cisplatin to neuronal cells has also been confirmed and recognized (Troy et al., 2000). The toxicity of these two substances is mainly mediated by ROS (Ravi et al., 1995; Schubert et al., 1995; Troy et al., 2000). In addition, gut microbiota dysbiosis is found to be closely related to neurodegenerative diseases (Shen et al., 2017; Brown and Goldman, 2020).

Detection of OCTN1 expression in the brain indicates that EGT can penetrate the blood-brain barrier and enter the brain to play a role (Kaneko et al., 1980; Lamhonwah et al., 2008; Tang et al., 2018). EGT has been found to promote the proliferation and differentiation of neuronal cells and relieve depressive symptoms in mice at a reasonable daily intake level (120 mg EGT/100 g diet) (Nakamichi et al., 2016). Recently, a community-based cross-sectional in Singapore also found that eating mushrooms (more than 2 times a week) contributes to improving the cognitive level and extending the life of patients (Feng et al., 2019). However, in the elderly, especially in patients with neurodegenerative diseases, the level of EGT is significantly decreased in the brain and plasma (Cheah et al., 2016a). All these findings hint that supplementation of EGT might be necessary to maintain neuronal cells function and prevent its neurodegeneration to some extent.

Studies of cells and animals that suffered neuronal injury confirmed that the potential action mechanisms of EGT for neurodegenerative diseases should be multiple. Firstly, EGT could directly inhibit the accumulation of bacteria and Aβ in the hippocampus and lipid peroxidation in neuronal cells (Yang et al., 2012). Secondly, EGT could affect other antioxidants, such as maintaining the GSH/GSSG ratios and the superoxide dismutase activity, and restore acetylcholinesterase activity in the brain (Song et al., 2010; Yang et al., 2012). Thirdly, EGT could prevent the formation of peroxynitrite (Jang et al., 2004). Thereby, it protects or decreases neuronal cells from Aβ-induced apoptosis and cisplatin-induced neuronal injury in a dose-dependent manner (Jang et al., 2004; Song et al., 2010; Yang et al., 2012). Furthermore, diabetes is closely related to neurodegeneration, and hyperglycemia induces neurotoxicity and neuronal cells apoptosis in the hippocampus (Grillo et al., 2003). It was found that EGT could directly reduce ROS levels and inhibit the transcription pathway of NF-κB, and it could also prevent the production of proinflammatory cytokines to inhibit neuroinflammation in the brain. Finally, EGT could protect neuronal cells from hyperglycemia-induced cytotoxicity (Song et al., 2017).

### 2.4 Other Diseases

Oxidative damage caused by overactivation of the immune response is the key pathogenic factor of chronic inflammation conditions, such as Crohn’s disease (CD), rheumatoid arthritis, and the inflammatory bowel diseases (Hussain et al., 2016; Cheah and Halliwell, 2020). In addition, mass spectrometry-based metabolomics studies found that abnormal lipid and amino acid metabolism, which associated with gut microbes, had also become the feature of inflammatory diseases (Lai et al., 2019; Lavelle and Sokol, 2020). At the same time, circulating EGT levels were detected to be significantly lower in CD patients than in healthy individuals, which seems to make it a potential biomarker for CD (Lai et al., 2019). One study showed that the functional expression of OCTN1 in the small intestine could promote the gastrointestinal absorption of EGT, thereby inhibiting intestinal inflammation (Shimizu et al., 2015), but whether this protective mechanism is related to gut microbiota has not been studied. Moreover, the gene *SLC22A4*, which encodes OCTN1, is located near the genes regulating inflammation, and it was found that the expression of OCTN1 mRNA in the immune cells was increased in some patients with chronic inflammation (Taubert et al., 2006, 2009). It is speculated that when inflammation occurs, the body may consciously cope with inflammation by catalyzing the expression of OCTN1 and increasing the level of EGT in immune cells, thereby exerting anti-inflammatory or antioxidant to prevent further aggravation of inflammation (Maeda et al., 2007; Cheah and Halliwell, 2020). However, Petermann et al. (2009) found another phenomenon that CD patients with variant OCTN1 L503F allele, which can improve the transportability of OCTN1, were more susceptible to mushrooms containing EGT. Mushroom intake showed lower beneficial effects on these patients, on the contrary, adverse effects accounted for a higher proportion (Petermann et al., 2009). This suggests that excessive intake of EGT may exhibit negative effects on some CD patients. Therefore, the beneficial effect of EGT may be related to its dose and its dose-effect should be investigated by more studies.

Pre-eclampsia is a hypertensive disease of pregnancy, which causes the placenta to be exposed to oxidative stress. Mitochondrial dysfunction is the main pathological feature of pre-eclampsia (McCarthy and Kenny, 2016). EGT treatment has been found to significantly improve certain phenotypic characteristics of pre-eclampsia and reduce the production of H_2_O_2_ in the mitochondria of the kidney (Williamson et al., 2020). The mechanism is that EGT directly reduces the production of mitochondrial ROS and improves the placental expression of ROS detoxification enzymes and its transcriptional regulators to improve mitochondrial function (McCarthy and Kenny, 2016; Kerley et al., 2018). This indicates that EGT may have a certain therapeutic potential in pre-eclampsia.

## 3 Advantages of L-Ergothioneine Over Other Antioxidants

The antioxidant activity of EGT has many advantages over other antioxidants such as GSH and ascorbic acid (Nielsen et al., 2015; Cheah and Halliwell, 2020). Firstly, as a natural antioxidant, EGT can accumulate millimolar concentrations in certain tissues without toxicity (Shires et al., 1997; Ey et al., 2007; Tang et al., 2018). According to the analysis of the source and use level of EGT, the combined intake of EGT is 1.7 mg/kg bw per day for adults and 3.7 mg/kg bw per day for children (Turck et al., 2016). High-dose or acute experiments in animals and cells did not show the toxic effects of EGT. For example, 5,000 μg/ml EGT did not induce genotoxicity and chromosomal aberration in the Chinese hamster lung cell study (Schauss et al., 2011). A large number of acute (2-weeks) or long-term (90-days) oral EGT supplementation (0.9% concentration) experiments conducted on Sprague-Dawley rats did not show reproductive and developmental toxicity (CiToxLAB France, 2012; CiToxLAB France, 2013a; CiToxLAB France, 2013b; Forster et al., 2015; Turck et al., 2016); even if the rats were orally administered to their maximum dose (1,600 mg/kg/d), and no adverse reactions occurred (Schauss et al., 2011; Nielsen et al., 2015; Marone et al., 2016). The EFSA has also provided the intake and safety assessment of synthetic EGT in supplementary diets, considering that it is safe under the recommended maximum intake (daily intake of 2.82 mg/kg bw for infants, 3.39 mg/kg bw for young children, and 1.31 mg/kg bw for adults including pregnant and lactating women) (Turck et al., 2017). Secondly, EGT can be ingested through diet and quickly distributed in most tissues by the specifical transportation of OCTN1 (Gründemann et al., 2005; Nakamura et al., 2008). At the same time, in comparison with the rapid metabolism of other antioxidants, EGT maintains a low level of metabolism and a high level of accumulation in the body (Gründemann, 2012; Cheah et al., 2017; Borodina et al., 2020). Thirdly, under physiological conditions, EGT mainly exists in the form of thione, which renders it ideal thermal stability and pH stability. Therefore, it does not auto-oxidize and does not promote the Fenton reaction of H_2_O_2_ with ferrous ions (Hand et al., 2005; Cheah and Halliwell, 2012; Cheah et al., 2016b; Cheah and Halliwell, 2021). In addition, previous studies also found that EGT had other biological functions, such as directly inhibiting the replication of certain viruses (Xiao et al., 2006), participating in erythrocyte proliferation and energy regulation (Kawano et al., 1982a; Dransfeld et al., 2005), catalyzing carboxylation or decarboxylation reactions (Brummel, 1989), regulating histamine and thyroid effects (Astwood and Stanley, 1947). However, whether these physiological functions play certain auxiliary roles in better exerting the protective effect of EGT merits further exploration.

## 4 Conclusion and Perspectives

To summarize, more and more *in vitro* and *in vivo* experiments proved that the antioxidant function of EGT was superior to some other natural antioxidants. As a non-toxic natural antioxidant, the antioxidant function of EGT makes it have the huge therapeutic or preventive potential for many oxidative stress-mediated diseases. Therefore, synthetic EGT has gradually attracted people’s attention and has been widely used in the food and cosmetics industry (Borodina et al., 2020; Han et al., 2021).

However, many unresolved questions may limit its further application in the prevention and treatment of diseases. Firstly, although there are many associations between dietary mushrooms and diseases (Martel et al., 2017; Wong et al., 2017), in addition to EGT, mushrooms also have other possible beneficial ingredients which have been shown to have certain antioxidant and anti-inflammatory activities (Muszyńska et al., 2018; Tsiantas et al., 2021). Table 1 summarized human studies on the possible associations of dietary mushrooms or EGT supplementation with health and diseases (Table 1), and more *in vivo* and clinical studies concerning the prevention or treatment of oxidative stress-related diseases by EGT supplements are strongly encouraged. Secondly, there are no substantial research on the correlation between the therapeutic dose of EGT and diseases. Thirdly, the expression level of OCTN1 plays an important role in the intake and accumulation of EGT and the exertion of its function in different tissues and cells (Kato et al., 2010; Sugiura et al., 2010; Tang et al., 2018). Therefore, it is of significance to pay much attention to the regulation of OCTN1 expression. Furthermore, there should be other undiscovered action mechanisms of EGT, which may complement its antioxidant function. For instance, the therapeutic benefits of many natural antioxidants were reported to be associated with their effects to modulate gut microbiota (Shen and Ji, 2019; Sun et al., 2021), while there are few studies on the influence of EGT on gut microbiota at present (Cheah and Halliwell, 2021). Therefore, it is of interest to further study the modulate effect of EGT on gut microbiota, which may potentially reveal other benefits of EGT. Understanding these mechanisms will provide important implications for future rational application of EGT.

**TABLE 1:** Summary of human survey studies on the association of ergothioneine or mushrooms with health and diseases.

Authors	Types of subjects	Meal planning	Detection indicators	Main outcomes
Calvo et al. (2016)	37 patients with early diabetes	100 g/d for 16 weeks *Agaricus bisporus* (EGT 3.2 mg/100 g)	The serum of EGT, some specific protective and oxidative stress biomarkers	Frequent consumption of mushrooms in patients with diabetes may yield potential anti-inflammatory and antioxidant health benefits
Cheah et al. (2017)	45 healthy young male volunteers	5 or 25 mg/d for 1 week EGT	The blood and urine level of EGT and some oxidative damage biomarkers	Supplemental EGT could be strongly absorbed and retained by the body, and at the same time, the oxidative damage and inflammatory biomarkers showed a significant decrease trend
Feng et al. (2019)	663 volunteers aged 60 and above	Number of times mushrooms are eaten per week	Correlation between mushroom consumption and mild cognitive impairment (MCI)	Mushroom consumption (more than 2 times a week) may reduce the odds of developing MCI.
Cheah et al. (2016a)	Aged Asian population over 60 with MCI and healthy	Normal diet	Relationship of EGT whole blood level with age and MCI	The low blood EGT levels may be a risk factor for neurodegeneration in the elderly
Smith et al. (2020)	3,236 volunteers	Normal diet	112 plasma metabolites	The high level of EGT may signal a lower risk of developing cardiometabolic disease and lower mortality
Petermann et al. (2009)	449 patients with CD and 370 controls	Normal diet	Correlation between genotype and dietary	OCTN1 variant single nucleotide polymorphisms may increase the risk of adverse symptoms associated with mushroom consumption
